# IRAK3-mediated suppression of pro-inflammatory MyD88/IRAK signaling affects disease severity in acute pancreatitis

**DOI:** 10.1038/s41598-023-37930-3

**Published:** 2023-07-04

**Authors:** Franziska G. Thiel, Saeedeh Asgarbeik, Juliane Glaubitz, Anika Wilden, Markus M. Lerch, Frank Ulrich Weiss, Matthias Sendler

**Affiliations:** Department of Medicine A, University Medicine, University of Greifswald, Fleischmannstr. 41, 17475 Greifswald, Germany

**Keywords:** Gastroenterology, Pancreatitis

## Abstract

Acute pancreatitis (AP), which is characterized by self-digestion of the pancreas by its own prematurely activated digestive proteases, is a major reason for hospitalization. The autodigestive process causes necrotic cell death of pancreatic acinar cells and the release of damage associated molecular pattern which activate macrophages and drive the secretion of pro-inflammatory cytokines. The MYD88/IRAK signaling pathway plays an important role for the induction of inflammatory responses. Interleukin-1 receptor associated kinase-3 (IRAK3) is a counter-regulator of this pathway. In this study, we investigated the role of MYD88/IRAK using *Irak3*−/− mice in two experimental animal models of mild and severe AP. IRAK3 is expressed in macrophages as well as pancreatic acinar cells where it restrains NFκB activation. Deletion of IRAK3 enhanced the migration of CCR2^+^ monocytes into the pancreas and triggered a pro-inflammatory type 1 immune response characterized by significantly increased serum levels of TNFα, IL-6, and IL-12p70. Unexpectedly, in a mild AP model this enhanced pro-inflammatory response resulted in decreased pancreatic damage, whereas in a severe AP model, induced by partial pancreatic duct ligation, the increased pro-inflammatory response drives a severe systemic inflammatory response syndrome (SIRS) and is associated with an increased local and systemic damage. Our results indicate that complex immune regulation mechanism control the course of AP, where moderate pro-inflammation not necessarily associates with increased disease severity but also drives tissue regenerative processes through a more effective clearance of necrotic acinar cells. Only when the pro-inflammation exceeds a certain systemic level, it fuels SIRS and increases disease severity.

## Introduction

Acute pancreatitis (AP) is the gastrointestinal disorder with the highest rate of hospital admissions among all non-malignant GI diseases. While 80% of patients have a self-limiting mild course of the disease, in 20% the disease takes a more severe course, characterized by systemic complications such as organ failure or infection of pancreatic necrosis. The major driving factor of severity is an exaggerated immune response^1–4^. Development of a systemic inflammatory response syndrome (SIRS) is common in severe AP patients. Macrophages/monocytes are the most numerous cells of the immune system which migrate into the inflamed organ^3,5^ and get activated by cytokines or damage associated molecular patterns (DAMPs) that are released by damaged acinar cells. The release of other cytokines, such as TNFα^3,6^, amplifies acinar cell damage and creates a self-fuelling cycle of necrosis driven inflammation.

Toll-like receptor (TLR) mediated activation of immune cells is mainly mediated by the MYD88/IRAK/NFκB cascade^7^. IRAK3 is an endogenous counter regulator of the TLR/MYD88 signalling, in order to prevent hyperinflammation^8^. TLRs detect a variety of pathogenic signalling molecules like bacterial lipopolysaccharides (LPS). Remarkably, AP is a primarily sterile inflammation, but beside PAMPs (pathogen associated molecular pattern) TLRs are also able to recognise multiple DAMPs (damage-associated molecular pattern) such as free DNA, free ATP or intracellular proteins released by necrotic cells^9,10^. Necrotic cell death is the dominant form of cell death during pancreatitis^11^. A second sensor axis for the detection of damage is the IL-1 Receptor signalling, which also involves the MYD88/IRAK/NFκB cascade. The IL-1 receptor family detects cytokines of the IL-1 cytokine family including “alarmins” like IL-1α or IL-33 which are also released during necrotic cell death^12^.

The severity of the AP course is believed to depend on the strength of the induced immune response which determines the course and prognosis of the disease. The TLR/IL-1R signalling pathway plays a critical role in translating local damage into a systemic immune response^13^. The aim of this study was to investigate the regulation of the immune response in relation to the role of the endogenous inhibitor IRAK3 in two mouse models of mild and severe acute pancreatitis.

## Results

### AP causes activation of the MYD88/IRAK3 signalling pathway in macrophages

**Figure 1:**
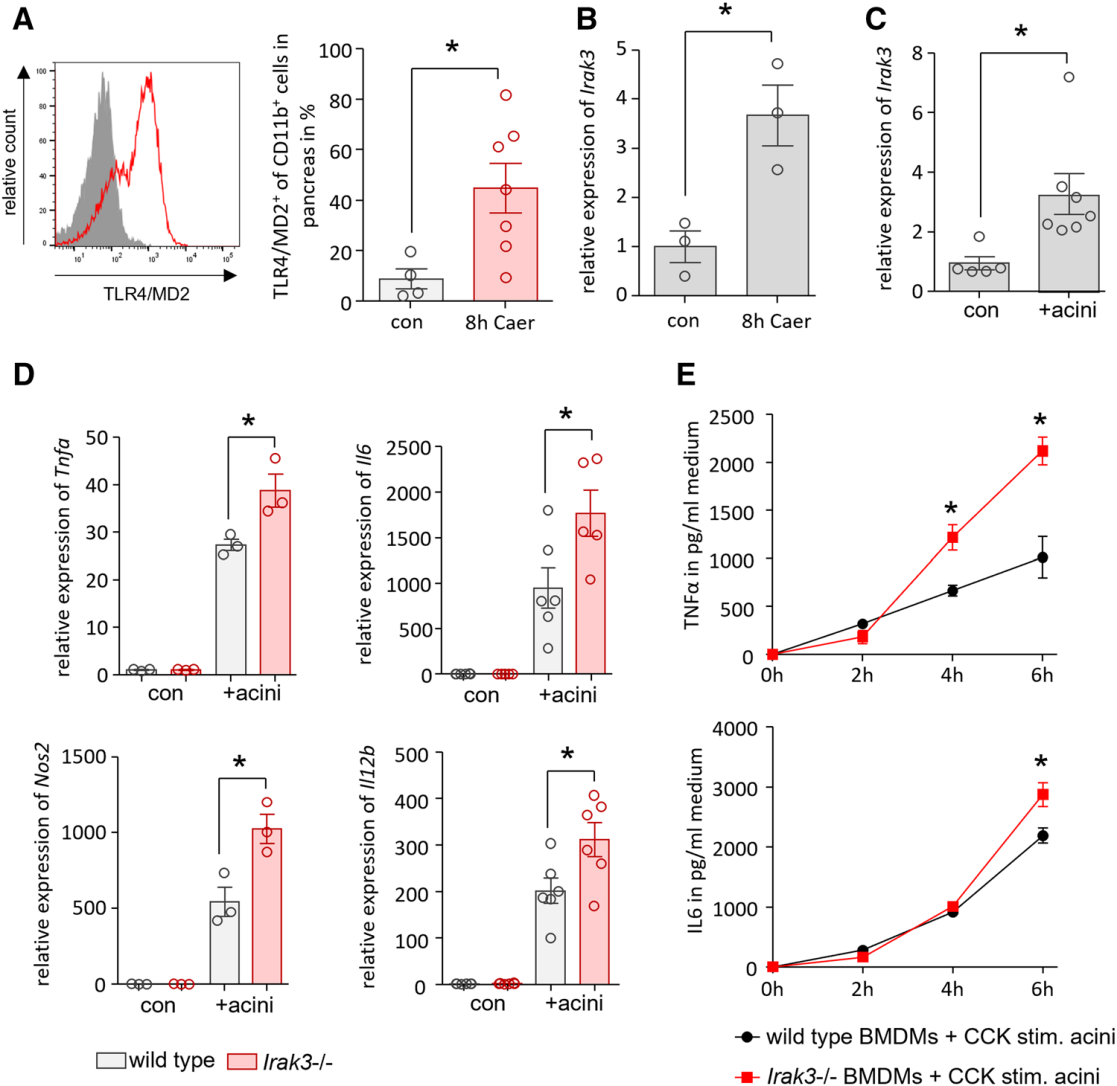
AP induction leads to activation of the MYD88/IRAK3 signalling pathway in macrophages. AP was induced by hourly injection of Caerulein (Caer) over a period of 8 h. (**A**) Flow cytometry of isolated leukocytes from pancreas showed a significant increase of TLR4/MD2^+^ CD11b^+^ cells (con n = 4, 8 h Caer n = 7). (**B**) qRT PCR showed a significantly increased expression of the *Irak3* gene (n = 3). (**C**) BMDMs co-incubated for 6 h with freshly prepared acini showed also a significantly increased expression of the *Irak3* gene (con n = 5 + acini n = 7). (**D**) Co-incubation of BMDMs from wild type and IRAK3 deficient mice with freshly prepared acini for 6 h showed a significantly increased expression of pro-inflammatory genes, such as *Tnfa* (n = 3), *Il6* (n = 6), *Nos2* (n = 3) and *Il12b* (n = 6). (**E**) Secretion of the pro-inflammatory cytokines IL-6 and TNFα in supernatant was also increased in IRAK3 deficient BMDMs (n = 4). Significant differences were tested using student’s t-test, all error bars indicate SEM. Significance level *p* < 0.05 is marked by an asterisk.

AP was induced in C57BL/6 mice by hourly injections of caerulein. Analysis of AP-induced TLR4 signalling in CD11b^+^ cells, which were isolated from murine pancreatic tissue, showed significantly increased labelling of the TLR4/MD2 complex (Fig. 1A). PCR analysis of the *Irak3* gene, encoding the IRAK3 protein, showed in pancreatic tissue a significantly increased mRNA level 8 h after the onset of the disease (Fig. 1B). IRAK3 is mainly expressed in macrophages where it acts as a negative regulator of TLR and IL1R signalling pathways. Co-incubation of bone marrow derived macrophages (BMDMs) with acinar cells, previously stimulated for 30 min with 0.001 mM CCK, showed a significantly increased *Irak3* gene expression in macrophages in response to their exposition to damaged acini (Fig. 1C). In a Co-incubation experiment of CCK-stimulated acinar cells with BMDMs from IRAK3 deficient mice we saw a significant higher expression of pro-inflammatory genes, such as *Tnfa*, *Il6*, *Nos2* and *Il12b* which are under the transcriptional control of NFκB (Fig. 1D). Also, the co-incubation with CCK-stimulated acinar cells caused a significantly increased release of TNFα and IL-6 into the medium (Fig. 1E).

### The AP-induced systemic immune response is increased in IRAK3 deficient mice

**Figure 2:**
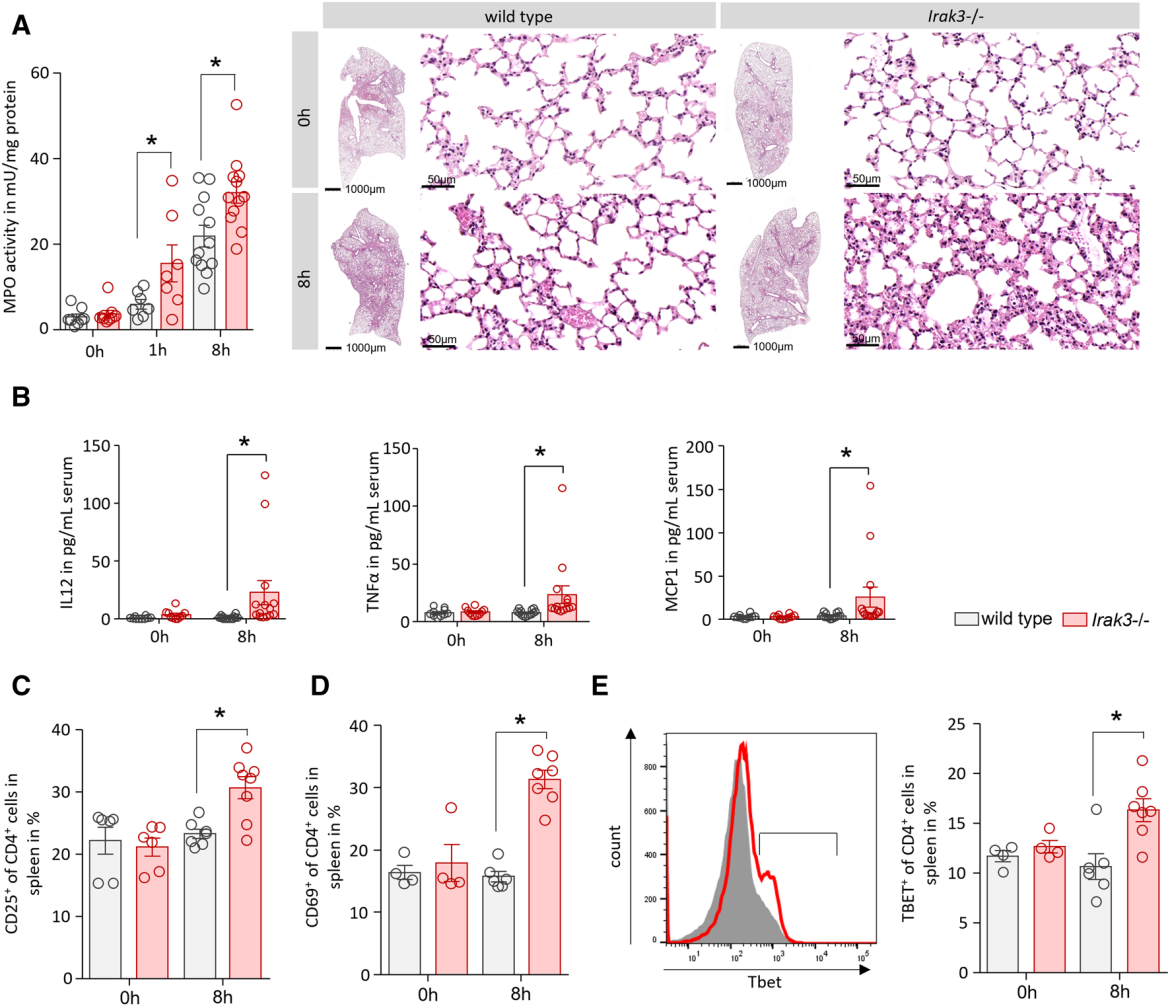
The AP-induced systemic inflammation is elevated in IRAK3 deficient mice. AP was induced by hourly injection of Caerulein (Caer) over 8 h. (**A**) Myeloperoxidase activity in lung tissue as well as H&E histology of lung sections showed a higher lung damage in *Irak3*−/− mice (n = 7–12 mice). (**B**) Serum cytokine and chemokine levels of IL-12p70, TNFα and MCP-1 were significantly elevated in *Irak3*−/− 8 h after onset of pancreatitis (n = 10–15 mice). (**C-E**) Flow cytometry of splenocytes showed a significantly increased T-helper cell activation, marked by CD25^+^/CD4^+^, CD69^+^/CD4^+^ and TBET^+^/CD4^+^ Th1 cells (n = 4–8 mice). Significant differences in lung MPO were tested using ANOVA followed by Bonferroni multiple comparison test, all other significant differences were tested using student’s t-test. Error bars indicate SEM. Significance level *p* < 0.05 is marked by an asterisk.

AP was induced by hourly injections of caerulein in wild type and *Irak3*−/− mice. We valuated myeloperoxidase activity in lung tissue as an indicator of a systemic immune response and found a significant increase in the IRAK3 deficient mice. H&E staining confirmed the involvement of the respiratory tissue in the immune response (Fig. 2A). The levels of pro-inflammatory cytokines IL-12p70 and TNFα, as well as the chemokine MCP-1 were significantly elevated in IRAK3 deficient mice, (Fig. 2B). IL-12p70 is known to induce in T-cells a Th1-cell differentiation. Flow cytometry analysis of splenocytes from these mice showed a significantly increased activation of CD4^+^ T-helper cells in *Irak3*−/− mice after induction of AP. The cell surface expression of T-cell activation markers CD25 and CD69 were significantly increased in *Irak3*−/− 8 h after onset of pancreatitis (Fig. 2C,D). Also, a significantly induced differentiation of T-cells towards a pro-inflammatory Th1 phenotype could be observed by intracellular labelling of the transcription factor TBET (Fig. 2E).

### Knockout of IRAK3 improves the outcome of AP

**Figure 3:**
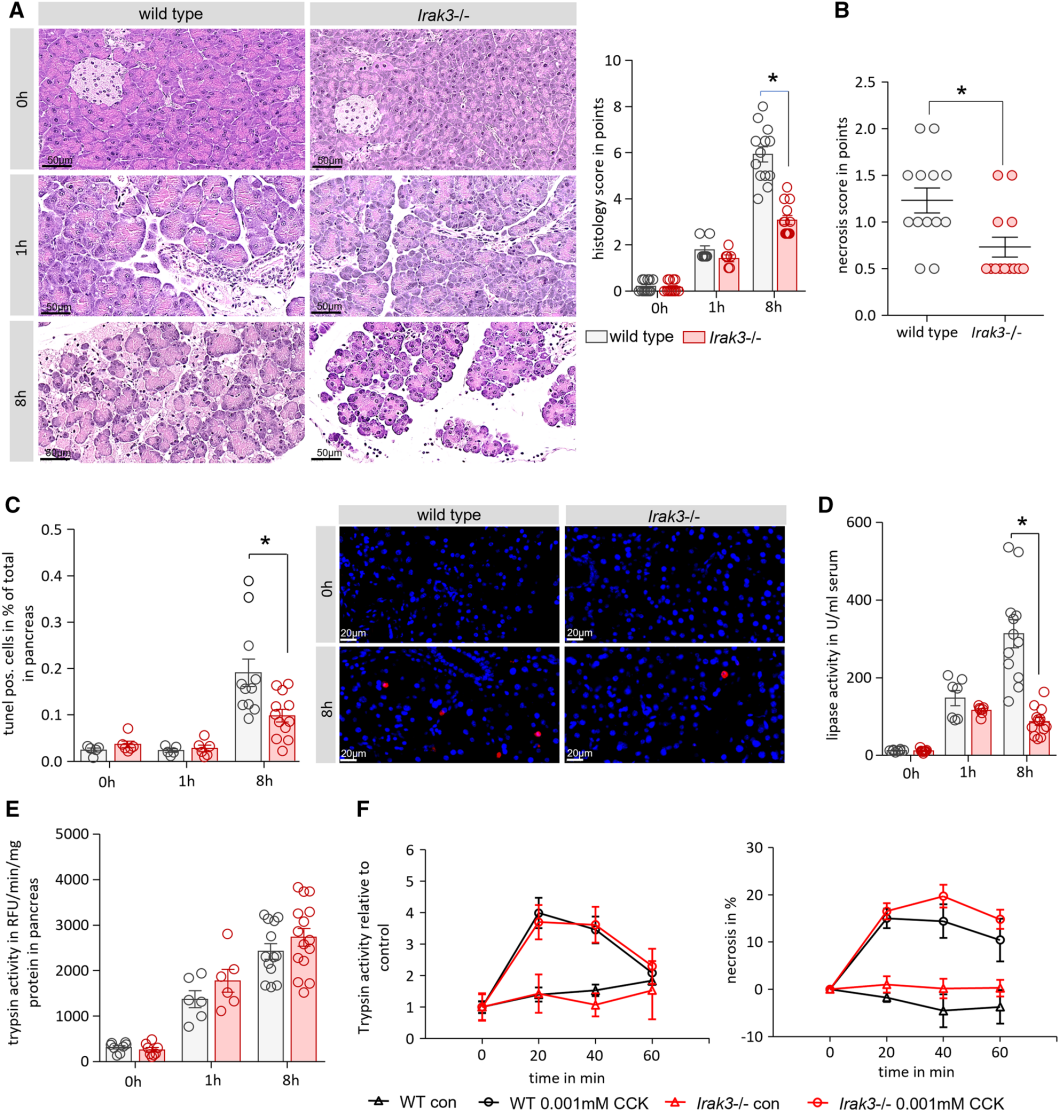
Knockout of Irak3 mitigates the pancreatic damage. AP was induced by hourly injection of Caerulein (Caer) over 8 h. (**A**) Scoring of H&E-stained pancreatic tissue sections showed significantly less tissue damage in *Irak3*−/− mice (n = 10–15 mice), (**B**) especially the number of necrotic cells was reduced in *Irak3*−/− mice. (**C**) Cell apoptosis, measured by TUNEL assay, and counting of TUNEL^+^ cell nuclei, also showed decreased numbers (n = 6–12 mice), (**D**) and the activity of serum lipase was also significantly reduced in *Irak3*−/− animals. (**E**) Trypsin activity was measured in pancreas homogenate and normalized to protein content (n = 6–15 mice). (**F**) Intracellular trypsin activity and necrosis ratio in response to 0.001 mM CCK was determined in living acinar cells over 60 min. No differences between wild type and *Irak3*−/− mice could be observed (n = 5). Significant differences for histology score, TUNEL, lipase activity and trypsin activity were tested using ANOVA followed by Bonferroni multiple comparison test, all other significant differences were tested using student’s t-test, all error bars indicate SEM. Significance level *p* < 0.05 is marked by an asterisk.

Analysis of pancreas histology showed a significantly reduced pancreatic damage in the IRAK3 deficient animals 8 h after onset of the disease (Fig. 3A), and especially the number of necrotic acinar cells was significantly reduced. (Fig. 3B). Beside necrotic cell death, also the number of apoptotic acinar cells was significantly reduced in the IRAK3 deficient mice, as was shown by the reduced number of TUNEL (TdT-mediated dUTP-biotin nick end labelling) positive cells in the pancreas (Fig. 3C). Serum Lipase activity, a biomarker of pancreatic damage, was significantly reduced in the *Irak3*−/− mice and confirmed our histological observations (Fig. 3D). Intrapancreatic trypsinogen activation was not different between wild type and IRAK3 deficient mice during the time course of caerulein induced pancreatitis (Fig. 3E). To investigate a direct effect of the *Irak3* knockout on the acinar cell response to caerulein, we isolated acinar cells from wild type and IRAK3 deficient mice, stimulated them with 0.001 mM CCK for 0 min, 20 min, 40 min and 60 min and measured intracellular trypsinogen activation, as well as necrotic cell death development by PI uptake. Acinar cells from both mouse strains responded in the same way to CCK (Fig. 3F).

### The local pancreatic immune response is elevated in IRAK3 deficient mice

**Figure 4:**
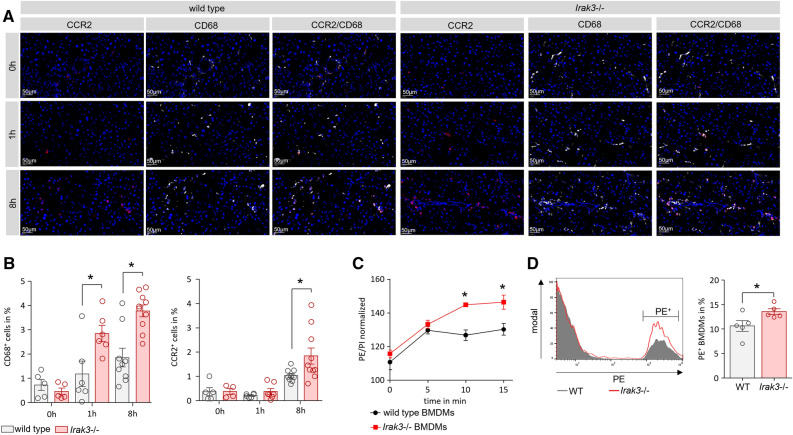
The AP-associated local immune response is elevated in *Irak3−/−* mice. (**A**) Labelling of nuclei by DAPI (blue), CD68 (white) and CCR2 (red) showed an increased number of macrophages in the pancreas of *Irak3*−/− mice. (**B**) Quantification of CD68 and CCR2 labelling shows significantly increased infiltration of CCR2^+^ cells as well as CD68^+^ cells. IRAK3 is mainly expressed in cells of myeloid origin, especially macrophages (n = 5–10 mice). (**C, D**) BMDMs of IRAK3 deficient mice showed a higher phagocytosis ratio compared to wild type BMDMs (n = 3). The uptake of PE marked beads was measured by a fluorometric reader as well as by flow cytometry (n = 5). Significant differences of CD68^+^ and CCR2^+^ cell counts were tested using ANOVA followed by Bonferroni multiple comparison test, all other significant differences were tested using student’s t-test, all error bars indicate SEM. Significance level *p* < 0.05 is marked by an asterisk.

To evaluate the local pancreatic immune response during pancreatitis, we labelled tissue sections with CD68 and CCR2 to identify pancreatic macrophages and infiltrating monocytes. Here we observed a significantly increased number of CD68^+^ macrophages in the pancreas of IRAK3 deficient mice after induction of AP (Fig. 4A,B). These cells were mainly positive for the chemokine receptor CCR2, which binds MCP-1 and recruit’s monocytes to the site of inflammation. It is known that pro-inflammatory macrophages can contribute to the pancreatic damage via the release of TNFα^3,6,14^, but we observed significant less acinar cell necrosis (Fig. 3A,B). Phagocytosis assays with bone marrow derived macrophages from wild type and *Irak3*−/− mice showed a higher phagocytosis ratio in the IRAK3 deficient cells (Fig. 4C,D) which suggests a faster clearance of pancreatic necrosis in the *Irak3*−/− mice.

### IRAK3 mediated TLR signalling is active in pancreatic acinar cells

**Figure 5:**
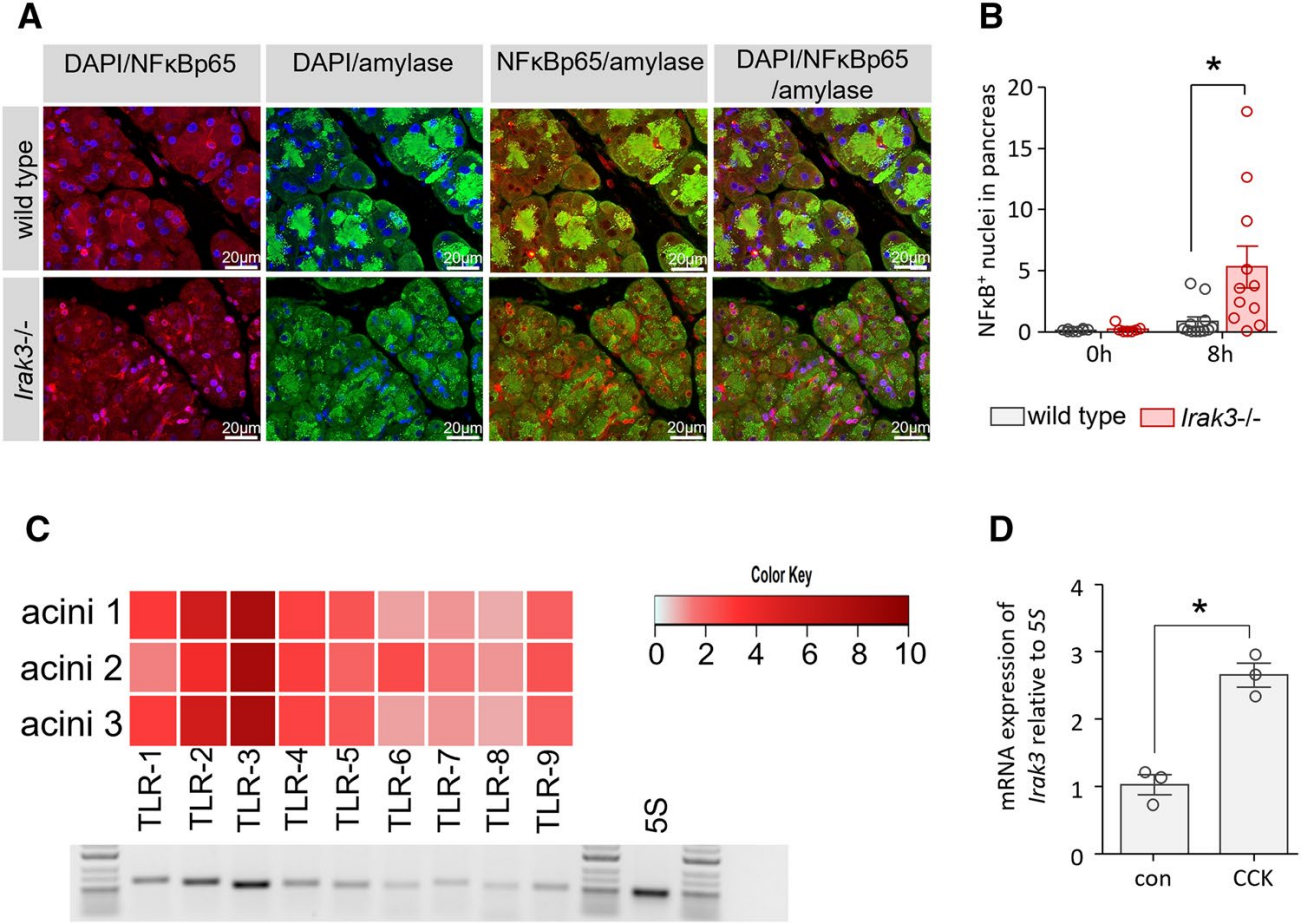
The AP-triggered NFκB activation in *Irak3−/−* mice is enhanced. (**A, B**) Immunofluorescent labelling showed 8 h after onset of pancreatitis an increased nuclear redistribution of NFκB in *Irak3*−/− mice compared to wild type animals (n = 10–12 mice). (**C**) PCR confirmed an expression of Toll-like receptors in isolated pancreatic acinar cells of wild type mice, especially TLR2, 3, 4 and 9. Heatmap illustrates the mRNA expression level of the TLRs of freshly prepared acinar cells (n = 3), compared to each other. Increasing red colour indicates a higher expression level of the specific TLR (n = 3). Representative Agarose gel illustrates the expression level of the TLRs. (**D**) Also the expression of *Irak3* could be observed in pancreatic acinar cells, mRNA levels showed a significant increase of *Irak3* after 2 h of stimulation with 0.001 mM CCK (n = 3). Significant differences were tested using student’s t-test, all error bars indicate SEM. Significance level *p* < 0.05 is marked by an asterisk.

We observed less tissue damage and a diminished disease severity in Irak3−/− mice in the presence of an increased pro-inflammatory immune response, which suggests a protective role of the MYD88/IRAK/NFκB pathway. Investigation of NFκB activation in pancreatic tissue showed significantly increased nuclear translocation of NFκBp65 in the *Irak3*−/− mice (Fig. 5A,B). In the next step we analysed Toll-like receptor expression in pancreatic acinar cells. Especially the expression of *Tlr2*, *Tlr3*, *Tlr4* and *Tlr9* mRNAs could be detected in acinar cells of wild type mice (Fig. 5C). Also, counter regulating IRAK3 could be detected in isolated acinar cells and showed an increased expression 2 h after stimulation with 0.001 mM CCK (Fig. 5D). These data make a direct effect of the IRAK3 deficiency on acinar cells likely. It is known that increased NFκB activation in pancreatic acinar cells leads to an elevated anti-stress response and ameliorates disease severity^15^.

### In a severe AP model a higher local pro-inflammatory response causes increased local damage

**Figure 6:**
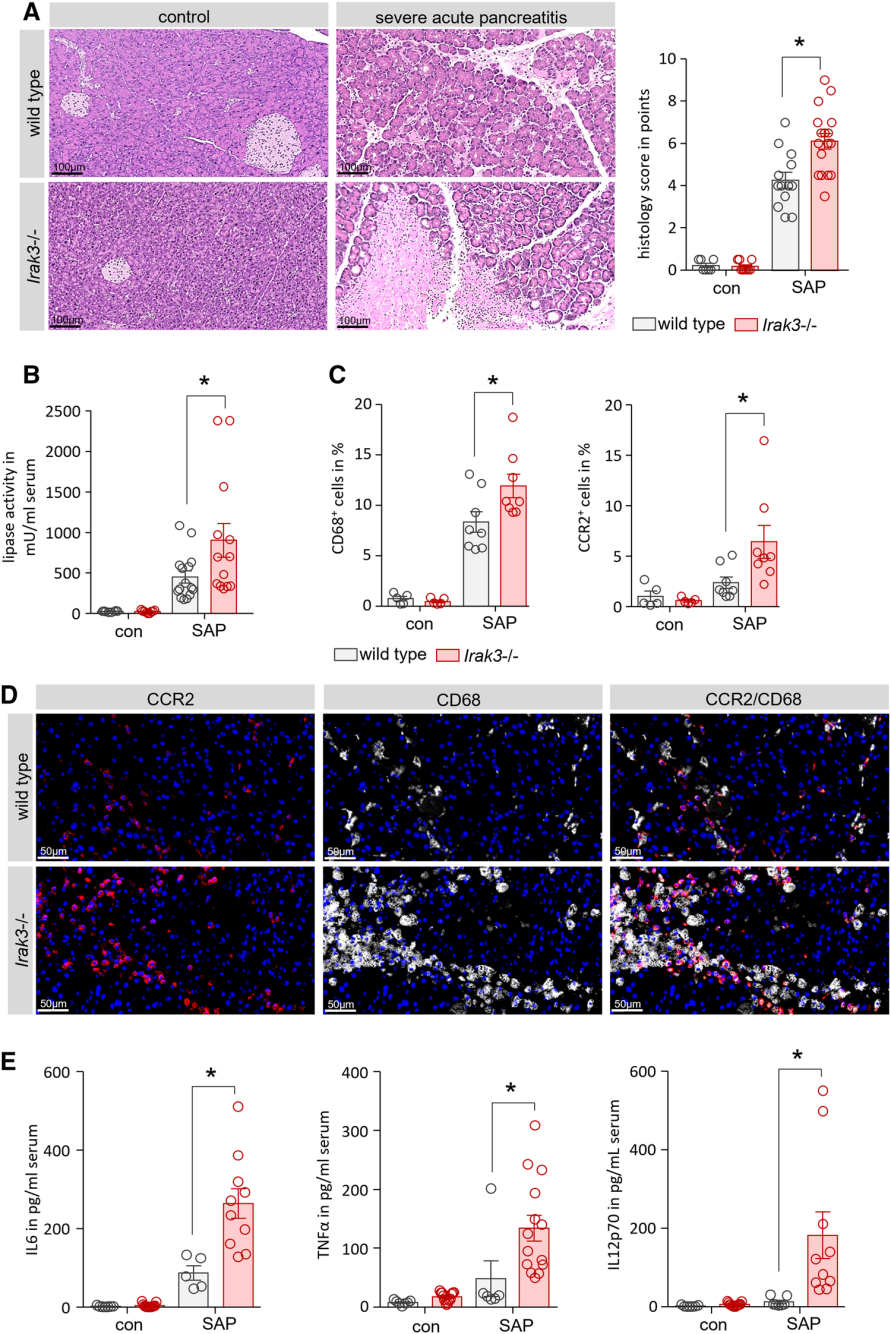
Deficiency of IRAK3 aggravates the outcome of severe acute pancreatitis. Severe acute pancreatitis (SAP) was induced by partial pancreatic duct ligation in *Irak3*−/− and wildtype mice. (**A**) 3d after onset of pancreatitis, *Irak3*−/− mice showed significantly increased pancreatic tissue damage by scoring of H&E stained sections (n = 10–18 mice). (**B**) Measurements of serum lipase activity showed also a significant increase in *Irak3*−/− mice (n = 10–18 mice). (**C, D**) Immunofluorescence labelling of CD68 and CCR2 in pancreatic tissue showed a significantly increased number of CD68^+^ and CCR2^+^ macrophages in IRAK3 deficient mice (n = 5–8 mice). (**F**) Also, serum cytokine levels of IL-6, TNFα and IL-12p70 were increased in *Irak3*−/− mice 3d after onset of pancreatitis (n = 6–15 mice). Significant differences were tested using student’s t-test, all error bars indicate SEM. Significance level *p* < 0.05 is marked by an asterisk.

To compare the results from the model of caerulein-induced mild pancreatitis with a more severe disease model, we induced AP by partial ductal ligation in mice. This model of AP causes a severe necrotizing form of pancreatitis^5,16^, associated with infected necrosis^4^ and organ damage^2^. H&E staining of pancreatic tissue sections demonstrated the severe necrotizing pancreatitis. In contrast to the model of caerulein induced pancreatitis, the degree of tissue damage was significantly higher in *Irak3*−/− mice compared to wild type mice (Fig. 6A). Measurement of serum lipase activity confirmed this observation and was significantly increased in *Irak3*−/− mice (Fig. 6B). The numbers of CD68^+^ and CCR2^+^ cells in pancreas were also increased in the IRAK3 deficient mice, which suggest a more prominent local inflammatory response in the absence of IRAK3 (Fig. 6C,D). The overall response is comparable but stronger than in the caerulein induced AP (Fig. 4A,B). Measurement of serum cytokine levels of IL6, TNFα and IL12p70 were significantly higher in the *Irak3*−/− mice (Fig. 6E).

### Knockout of IRAK3 increases the systemic inflammation in a severe AP model

**Figure 7:**
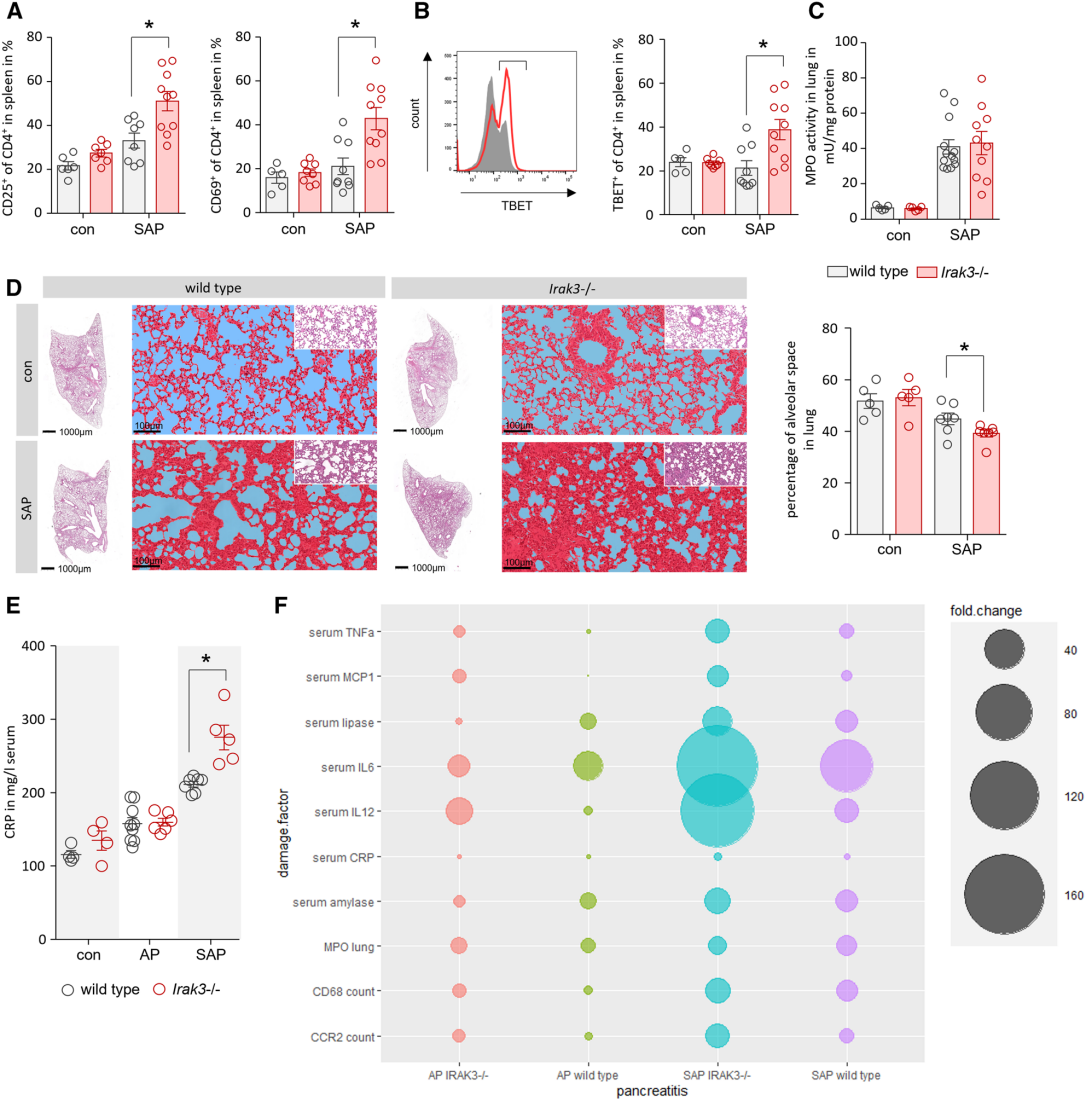
*The intensity level of the pro-Inflammation reaction determines the disease severity.* The systemic proinflammatory response was elevated in IRAK3 deficient mice compared to wild type animals. (**A**) Flow cytometry of isolated splenocytes showed a significantly increased expression of the cell surface markers CD25 and CD68 on CD4^+^ T-cells (n = 5–10 mice). (**B**) T-cell differentiation showed an elevated number of TEBT^+^ Th1-cells in spleen of *Irak3*−/− mice (n = 5–10 mice). (**C**) Measurement of MPO activity in lung tissue showed no differences between the mice lines (n = 5–10 mice). (**D**) Histological evaluation of lung tissue sections revealed a significant reduction of the alveolar space in *Irak3*−/− mice (n = 5–10 mice). (**E**) Measurement of serum CRP showed a significant AP-induced increase in *Irak3*−/− in the SAP model, whereas CRP was not different in the mild AP model (n = 5–10 mice). (**F**) Comparison of damage and outcome parameters in *Irak3*−/− and wild type mice in caerulein induced AP vs. the partial duct ligation model. We found markedly increased pro inflammation associated with damage parameters in the SAP model, whereas the pro-inflammation in the mild model of caerulein induced pancreatitis did not significantly affect the disease severity. Significant differences were tested using student’s t-test, all error bars indicate SEM. Significance level *p* < 0.05 is marked by an asterisk.

The systemic inflammatory response was investigated by flow cytometry analysis of CD4^+^ T-cells in spleen. We observed in severe pancreatitis an increased T-cell activation marked by the elevated cell surface expression of CD25 and CD69 on T-helper cells. This T-cell activation was significantly higher in the IRAK3 deficient mice (Fig. 7A). Only the T-cells from the spleen of *Irak3*−/− mice showed a significantly elevated level of TBET^+^ Th1-cells, whereas wild type mice showed no such signs of a Th1 response (Fig. 7B). Measurements of MPO activity in lung revealed no differences between the mice strains (Fig. 7C). SAP induced via partial pancreatic duct ligation is associated with respiratory dysfunction which could be measured by quantification of the alveolar space in histological sections^2^. In contrast to the lung MPO data we observed a significantly reduced lung volume in IRAK3 deficient mice after induction of SAP (Fig. 7D). Measurement of the C-reactive protein (CRP) in serum, a clinical diagnostic marker of inflammation, showed no dramatic increase in caerulein induced pancreatitis. Notably, IRAK3 deficient mice showed significant higher serum levels of CRP in the model of partial duct ligation. High CRP levels indicate the induction of SIRS, which is known to associate with a more severe disease course (Fig. 7E). A comparative summary of both models shows an increased systemic and local pro-inflammatory response in general, but with different outcome concerning pancreatic damage. Whereas the milder inflammatory response does not elevate the disease severity in the model of caerulein induced AP, the markedly elevated inflammatory response in the partial pancreatic duct ligation model was associated with significantly increased disease severity (Fig. 7F). These results suggest a dose dependent disease driving mechanism of pro inflammation.

## Discussion

Acute pancreatitis is characterized by a pronounced pro-inflammatory immune response that may develop into systemic inflammatory response syndrome (SIRS), increased pancreatic damage and systemic complications such as organ failure^1,2,4,16^. A crucial role for the induction of the immune response play Toll-Like and IL-1 receptor-signalling pathways^13,17,18^. Both pathways activate the MYD88/IRAK cascade, which results in the activation of transcription factor NFκB^19^. MYD88 is present in a complex with IRAK1/2 and IRAK4 which transmits TRAF6-mediated activation of the transcription factor NFκB in a phosphorylation-dependent manner. IRAK3 is a counter regulator of this signalling pathway, IRAK3 prevents dissociation of the IRAK-TRAF6 complex from the myddosome and thus prevents phosphorylation of NFκB^8,20^. IRAK3 is mainly expressed in macrophages, and regulates cytokine production through its inhibitory function^21^.

During acute pancreatitis, a pronounced pro-inflammatory immune response can occur, which further increases the local organ damage^3,22^ and is responsible for the development of systemic complications^1,2,4^. Our results from the mild model of caerulein-induced pancreatitis in *Irak3*−/− animals show that a limited degree of pro-inflammation may however be beneficial for tissue integrity, by clearance of necrotic cells and damaged tissue, and is not necessarily associated with increased severity. It is known that a prolonged clearance of necrosis is associated with extended inflammation and impaired regeneration^23^. Despite increased macrophage infiltration of the pancreas and observable systemic immune responses, the local pancreatic damage was significantly lower in IRAK3 deficient mice. In addition to macrophages pancreatic acinar cells also express IRAK3 and a repertoire of Toll-like receptors that likewise stimulate NFκB activation in acinar cells. NFκB activation occurs rapidly in acinar cells and forms the cornerstone of the immune response during pancreatitis^24^. Notably, a pancreas-specific deletion of the RelA the p65 subunit of NFκB did not attenuate inflammation and reduce disease severity but resulted in dramatically increased pancreatic damage and pro-inflammation^25^. On the other hand, increased activation of RelA by deletion of the inhibitor IκBα was protective during AP^15^. NFκB regulates not only the expression of inflammatory genes, but also of various stress response and survival genes that are protective and limit the organ damage^15^. Following induction of mild AP we observed significantly increased NFκB nuclear localization in *Irak3*−/− mice indicating an elevated anti-stress response that can help to decrease acinar cell damage. In addition, macrophages of IRAK3 deficient mice showed a higher phagocytosis ratio, which suggests an accelerated clearance of necrotic cell debris in the pancreas.

The clearance of necrotic cells is a necessary first step for the initiation of tissue regeneration after pancreatitis. On the one hand, rapid phagocytosis of necrotic cells prevents the spread of necrosis^23,26^. Necrotic cells release high amounts of ROS, which in turn aggravates the tissue damage^26^. On the other hand, enhanced clearance limits the release of DAMPs, leading to a decreased polarization of surrounding macrophages towards a pro-inflammatory phenotype^5,27^. Thus, rapid clearance of tissue damage helps to prevent necrosis from spreading and may attenuate disease severity and progression. Tissue fibrosis is another major feature of regeneration after AP or acute relapse during chronic pancreatitis. In a necrosis/fibrosis sequence cleared necrotic tissue is replaced by extracellular matrix proteins and thus directly correlates the number of necrotic cells with the induced fibrogenesis^28,29^. Therefore, rapid clearance of necrosis can influence the subsequent regeneration process. However, IRAK3 is also involved in the type 2 immune response and can trigger fibrogenesis directly via the IL-33 ST2 axis on Th2 and ILC2 cells.^29,30^. The deletion of IRAK3 could therefore also have a direct effect on the tissue fibrosis.

As a second model of pancreatitis, we induced a more severe form of the disease by partial pancreatic duct ligation^2,4,5,16,28^. Here we observed an aggravating effect of the IRAK3 deletion, contrary to the mild model of caerulein induced pancreatitis. The model of SAP induced by partial duct ligation resulted in a prominent immune response, driven by pro-inflammatory macrophages^5,31^. In contrast to the caerulein induced pancreatitis, an increased anti-stress response cannot overcome the disease-causing blockade of the duct. Therefore, the activation of the MYD88/IRAK signalling pathway in immune cells can play a more prominent role, which triggers markedly elevated inflammatory markers and increased local and systemic damage. These results suggest a cell specific effect of the TLR/MYD88/IRAK pathway. Whereas the MYD88/IRAK pathway activates in acinar cells a damage-limiting anti-stress response^15,32^, the same pathway leads in cells of the innate immune system to an increased pro-inflammatory response^8^. In the severe model of AP, pro-inflammation reaches a systemic level, accompanied by a cytokine storm and severity driving SIRS, whereas a limited pathway activation enhances the clearance of pancreatic necrosis and does not necessarily result in a systemic hyperinflammation. Apparently, it is not only relevant in which cells NFκB is activated, but the level of activation in immune cells also plays an important role in determining the severity of pancreatitis. Our study demonstrated the complex nature of the immune response during AP and proves that a moderate pro-inflammation may be necessary to resolve the pancreatic damage. To investigate in more detail the cell specific influence of IRAK3 in acinar cells, macrophages or other immune cells, experiments in conditional knockouts using cell-specific promoter systems such as the Cre-Lox system would be helpful. This would allow us to distinguish between the TLR/MYD88/IRAK signalling effect of IRAK3 deficiency in pancreatic acinar cells and in myeloid cells like macrophages.

Beside the local pancreatic damage, AP induced in both models an increased systemic response. Lung MPO activity or decreased alveolar space could be observed in *Irak3*−/− mice as well as elevated serum levels of pro-inflammatory cytokines like IL6, which is known to be involved in pancreatitis mediated lung damage^33^. Recent data have shown that the deletion of TLR4 in the intestine leads to severe gut dysbiosis associated with increased disease severity during caerulein induced pancreatitis^34^. IRAK3 is known to influence the microbiome by regulating mucosal defence mechanisms^35^ and to prevent bacterial translocation into pancreatic necrosis^4^, the most significant risk factor for disease outcome^36^. These data suggest that in addition to the local immune response also the systemic immune reactions like in spleen^16^, duodenal mucosa^4^, lung^33^ and kidney^2^ affect disease severity and the prognosis of AP.

In conclusion, our data revealed the two faces of the pro-inflammatory immune response during AP. On the one hand increased NFκB activation in acinar cells strengthens anti-stress response mechanisms and ameliorates the local damage^15,25^. A moderate pro-inflammation also stimulates macrophages to clear necrotic areas. On the other hand strong NFκB activation in pancreatic macrophages results in uncoupled pro-inflammation characterized by SIRS and is associated with increased systemic damage^5,16,27^. Our study suggests that the systemic response is not a direct consequence of the local pancreatic damage but is rather determined by the (local) activation of immune cells.

## Material and methods

### Antibodies and reagents

Cholecystokinin CCK-8 (Sigma Aldrich, C2901), Collagenase of Clostridium histolyticum (EC.3.4.24.3) from (Serva, lot no. 14007), MCSF (BioLegend, Cat.576406), anti-NFκBp65 (Cell signaling, 8242), CD68 (antibody-online, ABIN181836), CCR2 (abcam, ab273050), TLR4/MD2 (BioLegend PE/Cy7 Cat. 117610), CD11b (BioLegend PerCP/Cy5.5 Cat.101228), CD69 (BioLegend BV510 Cat.104531), TBET (BioLegend PerCP/Cy5.5 Cat. 644805), CD25 BioLegend PE/Cy7 Cat. 102015), CD4 (BioLegend PE Cat. 100408), anti-α-amylase (Santa Cruz, sc-46657).

### Animal model

*Irak3*−/− mice (B6.129S1-*Irak3*^*tm1Flv*^/J) were obtained from JaxMice and were bred in the central animal facility of the university medicine Greifswald. C57BL/6-J mice were obtained from Charles River (Sulzfeld, Germany). All mice were maintained under pathogen-free conditions in ventilated animal cabinets with a 12 h light–dark cycle at a temperature of 21–24 °C (humidity 50–70%) and with access to food and water ad libitum. 8–12 weeks old male or female mice were used for the experiments. AP was induced by hourly intraperitoneal injections of caerulein (4030451, Bachem) (50 µg/kg/bodyweight) to a maximum of 8 h^3^. AP animals were sacrificed 1 h and 8 h after the first caerulein injection. SAP was induced by partial duct ligation followed by a single injection of caerulein 48 h after surgery, as previously described^2,4,5,16,28^. The mice were anesthetized with ketamine/xylazine, the peritoneal cavity was surgically opened, and the pancreas was exposed. A severe acute form of pancreatitis was induced by ligation of the pancreatic duct at the junction between the gastric and the duodenal lobe like previously described. The animals receive a single i.p. injection of caerulein (50 μg/kg/body weight) 48 h after surgical duct ligation. The mice were sacrificed 24 h after the caerulein injection (72 h after surgical duct ligation).

### Ethics declaration

All animal experiments were previously approved by an animal care committee (Landesamt für Landwirtschaft, Lebensmittelsicherheit und Fischerei Mecklenburg-Vorpommern LALLF-7221.3-1-011/17) and performed in accordance to the national guidelines for animal experiments. All animal experiments were performed in accordance to the ARRIVE guidelines.

### Isolation of bone marrow derived macrophages (BMDMs)

Myeloid progenitor cells were isolated from the bone marrow of mice (femur and tibia) and were cultivated in RPMI medium containing 5% fetal calf serum and PenStrep in the presence of 20 ng/mL macrophage colony-stimulating factor (M-CSF) for the differentiation into macrophages. Cells were maintained over 5–7 days until confluency. Macrophages were co-incubated with freshly prepared acini for 6 h. Phagocytosis was measured by Phagocytosis Assay Kit (IgG PE) from Cayman Chemicals (cat. 600540, Ann Arbor, MI USA), using a fluorometer (FLUOstar Optima from BMG Labtech, Ortenburg germany) or by flow cytometry (BD, *LSRII*).

### Isolation of pancreatic acinar cells

Acinar cells were freshly prepared by collagenase digestion under sterile conditions in the presence of PenStrep^3,37^. The pancreas was removed carefully and transferred into Dulbecco’s modified Eagle medium containing 2% bovine serum albumin and 10 mM HEPES in the presence of 1 mg collagenase and 1% PenStrep. Acinar cells were stimulated with 0.001 mM cholecystokinin (CCK) for 30 min and washed carefully before co-incubation with BMDMs. Intracellular enzyme measurement and necrosis detection was performed in living acinar cells. Cells were isolated as previously described and stimulated with CCK up to 60 min. Intracellular trypsin activity was measured against a fluorogenic substrate (R110-Ile-Pro-Arg) in a kinetic series for 60 min. Necrosis was determined by propidium iodide uptake. Both were measured by fluorometer (FLUOstar Optima from BMG Labtech, Ortenburg Germany) at 37 °C in medium (pH 7.4) containing 24.5 mm HEPES, 96 mm NaCl, 11.5 mm glucose, 6 mm KCl, 1 mm MgCl_2_ 6H_2_O, 0.5 mm CaCl_2_ 2H_2_O, 2.5 mm NaH_2_PO_4_ H_2_O, 5 mm sodium fumarate, 5 mm sodium glutamate, 5 mm sodium pyruvate, and 1% BSA and DMEM.

### Myeloperoxidase measurement

MPO activity was determined as described previously^3,5^. Lung tissue was homogenized in 20 mM potassium phosphate buffer (pH 7.4) on ice and centrifuged. The pellet, containing the neutrophils and monocytes, was resuspended in 50 mM potassium phosphate buffer (pH 6.0) containing 0.5% cetyltrimethylammoniumbromide. The suspension was frozen/thawed in 4 cycles, sonicated, and centrifuged at 20,000*g*. Myeloperoxidase activity was measured in 50 mM potassium phosphate buffer (pH 6) containing 0.53 mM O-dianisidine and 0.15 mM H_2_O_2_ as kinetic over time with a Spectramax (Molecular Devices, San Jose, CA USA) Spectrophotomenter. MPO activity was calculated against protein content of lung tissue homogenate.

### CRP and Cytokines determination in serum and supernatant

IL-12p70, IL-6, MCP-1, and TNFα were measured in serum samples of mice and cell culture supernatant by Cytometric Bead Array (CBA) Mouse inflammation kit (cat. 552,364 BD Bioscience, San Jose, CA USA). Concentration of C-reactive protein in serum was determined by Mouse C-Reactive Protein/CRP DuoSet ELISA (R&D Systems, Minneapolis, MN USA).

### Histology

Paraffin sections were used for hematoxylin and eosin staining. Slides were scanned and digitally stored with a slide scanner (Pannoramic MIDI BF/FL, Sysmex Norderstedt, Germany). Histology severity scoring was performed according to Niederau et al.^38^. Acinar cell necrosis, oedema, infiltration and vacuolization were quantified in a blinded manner. Quantification of alveolar space in lung slides was performed by Quant centre software from Sysmex as previously described^2^.

### Apoptosis assay

Quantification of apoptotic cells was performed by TUNEL assay (FragEL™ DNA fragmentation detection kit from Millipore, catalog no. QIA39–1EA) using paraffin sections. Slides were scanned with slide scanner (Pannoramic MIDI BF/FL, Sysmex Norderstedt, Germany). Quantification of apoptotic cells was performed by Quant centre software from Sysmex, TUNEL positive cells were calculated against total cell number^37^.

### Immunofluorescence labelling

Immunofluorescence labelling of CD68 and CCR2 was performed on 2 µm cryo slides, labelling of NFκBp65 and α-amylase was performed on 2 µm paraffin slides. The primary antibodies were used in a 1:100 dilution and incubated over night at 4 °C. The secondary antibodies were used in a 1:200 dilution, for 1 h at room temperature. Nuclear staining was performed by DAPI. Slides were scanned by slide scanner (Pannoramic MIDI BF/FL, Sysmex Norderstedt, Germany). Quantification of positive cells was performed by Quant centre software from Sysmex, positive cells were calculated against total cell number.

### Lipase activity

Activity of pancreatic lipase was determined as photometric assay (Photometer: Spectramax plus, molecular devices) in serum by P-Lip-Kit (cat. 03029590, Roche Diagnostic, Mannheim Germany) according to the manufacturers protocol.

### Trypsin activity

Trypsin activity was determined in pancreas homogenate. Protease activity measurement was determined as kinetic over 60 min at 37 °C. Activity was measured in buffer containing 100 mM TRIS and 5 mM CaCl_2_ and 10 µM of the fluorogenic substrate (R110 Ile-Pro-Arg)^37^. Trypsin activity was calculated against protein content.

### Flow cytometry

The Spleen was removed and homogenized directly after sacrificing the mice by using a 70 µm cell strainer. Splenocytes were washed with PBS and centrifuged at 300×*g* for 6 min. The cell pellet was resuspended in 1 mL erythrocyte lysis buffer, containing: 150 mM NH_4_Cl, 10 mM KHCO_3_, 10 mM EDTA ∙ 2Na. After 5 min the reaction was terminated by PBS.

Isolation of leukocytes from pancreatic tissue was performed by using Multi Tissue Dissociation Kit 1 (130-110-201, MiltenyiBiotec) as previously described^29^. The pancreas was homogenized with the gentleMACS Dissociator (130-093-235, MiltenyiBiotec) at 120 runs for 37 s. After incubation at 37 °C for 20 min under continuous rotation the samples were again dissociated at 168 runs for 37 s. The remaining acinar cells were removed by filtration through a 70 µm cell strainer. The cell suspension was centrifuged at 300×*g* for 6 min.

Cells were washed with FACS buffer (0.02% sodium azide, 2% FCS, 2 mM EDTA in 1 × PBS), 1 × 10^6^ cells per tube were pre-incubated with 1 µL FcR Blocking Reagent (130-092-575, MiltenyiBiotec) to block non-specific Fc-mediated interactions. In the next step, extracellular markers (1:50 CD4, CD69 and CD25; 1:50 CD11b, TLR4 MD2) were labelled by adding the antibody cocktail and incubated at 4 °C for 30 min. After fixation and permeabilization (Transcription Factor Staining Buffer Set, 130-122-981, Miltenyi Biotec; Fixation Buffer, 420,801, BioLegend) the cell suspensions were again treated with FcR Blocking Reagent and labeled with the intracellular antibody against the transcription factor TBET. Finally, the samples were analyzed by flow cytometry (BD, *LSRII*), the results were analysed by *FlowJo.*

### PCR and real time PCR

Total RNA was extracted from pancreas, acinar cells and BMDMs using TRIzol Reagent (15596026, life technologies). Samples were homogenized in 500 µL TRIzol and with a TissueLyser. After the addition of 100 µL Chloroform, samples were mixed and centrifuged at 22,000×*g*, 15 min at 4 °C. Subsequently, the RNA containing phase was transferred to a new tube. RNA was precipitated by adding 250 µL isopropyl alcohol. The sample was incubated for 10 min at RT and centrifuged at 22,000×*g*, 10 min, 4 °C. The pellet was washed with 500 µL 75% ethanol and again centrifuged at 6500×*g* for 10 min at 4 °C. After air-drying the pellet was solved in 100 µL A. dest.

RNA samples (2 µg) were transcribed into complementary DNA (cDNA). The cDNA was synthesized using a standard protocol: 2 µg RNA; 5 µM OligodT primers; 75 ng random primers; 0.5 µM dNTP Mix; 1 × First Strand Buffer (18080044, Invitrogen); 10 µM DTT; 40 Units RNasin Ribonuclease Inhibitor (N251B, Promega) and 200 Units M-MLV RT (28025013, invitrogen). The total volume per reaction was 20 µL.

The expression of genes of interest was analyzed by reverse transcription-quantitative PCR (RT-qPCR) using the SYBR-green method. The qPCR amplification was performed in a volume of 5 µL containing 1 × SYBR Green PCR Master Mix (4309155, applied biosystems), 300 ng gene-specific oligonucleotide primers (reverse and forward) and a 1:10 dilution of cDNA fragments in two technical replicates. Detectedtranscript levels were normalized to *Rn5s* and to the relative expression in control mice. Quantitative mRNA alterations were determined using the 2^−∆∆Ct^-method as previously described^4,29^.

### Primer

*Tlr1* forward 5′-GCTGGTGTTAGGAGATGCTTAT-3′ reverse 5′-GACGGACACATCCAGAAGAAA-3′, *Tlr2* forward 5′-TGAAGTCCAGCAGAATCAATACA-3′ reverse 5′-CCGAACCAGGAGGAAGATAAAC-3′, *Tlr3* forward 5′-GTGCATCGGATTCTTGGTTTC-3′ reverse 5′-GACCCAGTCTCTGTCTTTATGG-3′, *Tlr4* forward 5′-GCTTACACCACCTCTCAAACT-3′ reverse 5′-ACAGCCACCAGATTCTCTAAAC-3′, *Tlr5* forward 5′-GAAGACTGCGATGAAGAGGAAG-3′ reverse 5′-CAAGGGTGATGACGAGGAATAG-3′, *Tlr6* forward 5′-GGGTAAATCCTCCACCATTCAG-3′ reverse 5′-GCTAACATGAAGCCAGGTAGAG-3′, *Tlr7* forward 5′-AACCTTTCCCAGAGCATACAG-3′ reverse 5′-GGAGCCTCTGATGAGACAAATAA-3′, *Tlr8* forward 5′-CCTCTCTAAGGCTAGGGTAACT-3′ reverse 5′-TGCCCAGAAGACAGCATTT-3′, *Tlr9* forward 5′-TGGACGGGAACTGCTACTA-3′ reverse 5′-CAGAGACAGATGGGTGAGATTG-3′, *Irak3* forward 5′-GTGATCATGGAGGTTCTAACGG-3′ reverse 5′-GAGGACAGGGTGGTATCT-3′, *Nos2* forward 5′-TTGGAGCGAGTTGTGGATTTG-3′ reverse 5′-TAGGTGAGGGCTTGGCTGAG-3′, *Tnfa* forward 5′-GCCTCCCTCTCATCAGTTCTAT-3′ reverse 5′-CACTTGGTGGTTTGCTACGA-3′, *Il6* forward 5′- CCAGAGTCCTTCAGAGAGATACA-3′ reverse 5′-CCTTCTGTGACTCCAGCTTATC-3′, *Il12b* forward 5′-AGCACGGCAGCAGAATAAA-3′ reverse 5′-CTCCACCTGTGAGTTCTTCAAA-3′, *Rn5s* forward 5′-GCCCGATCTCGTCTGATCTC-3′ reverse 5′-GCCTACAGCACCCGGTATTC-3′.

### Software

Flow cytometry was analysed by FlowJo Software (BD Biosciences). All graphs were prepared by GraphPad Prism and SigmaPlot. Histology was evaluated by Quant centre software from Sysmex. Bubble chart was prepared by R Studio.

## Data Availability

All data generated or analysed during this study are included in this published article.
